# Health Education in Mass Gatherings: A Scoping Review to Guide Public Health Preparedness and Practice

**DOI:** 10.3390/healthcare13151926

**Published:** 2025-08-07

**Authors:** Rania Zaini, Altaf A. Abdulkhaliq, Saleh A. K. Saleh, Heba M. Adly, Salwa Abdulmajeed Aldahlawi, Laila A. Alharbi, Hani M. Almoallim, Nahla H. Hariri, Ismail Ahmad Alghamdi, Majed Sameer Obaid, Amar Mohammad A. Alkhotani, Aous Sami Hayat Alhazmi, Anas A. Khan, Fahad A. Alamri, Mohammed A. Garout

**Affiliations:** 1 Department of Community Medicine and Pilgrims Health Care, College of Medicine, Umm Al-Qura University, Makkah 24382, Saudi Arabia; 2 Department of Biochemistry, Faculty of Medicine, Umm Al-Qura University, Makkah 24382, Saudi Arabia; 3 Department of Basic and Clinical Oral Science, Faculty of Dental Medicine, Umm Al-Qura University, Makkah 24382, Saudi Arabia; 4 Department of Internal Medicine, Faculty of Medicine, Umm Al-Qura University, Makkah 24382, Saudi Arabia; 5 Medical Center, Umm Al-Qura University, Makkah 24382, Saudi Arabia; 6 Department of Emergency Medicine, College of Medicine, King Saud University, Riyadh 11421, Saudi Arabia; 7 Global Centre for Mass Gatherings Medicine, Ministry of Health, Riyadh 11176, Saudi Arabia; 8 Department of Community Medicine, Faculty of Medicine, Umm Al-Qura University, Makkah 24382, Saudi Arabia

**Keywords:** education, hajj, health, mass gathering, prevention

## Abstract

**Objectives**: In view of a lack of evidence on the subject, we aimed to perform a scoping review to understand the impact of health education among people attending mass gatherings. **Methods**: We followed the Preferred Reporting Items for Systematic Reviews and Meta-Analyses extension for Scoping Reviews (PRISMA-ScR) Guidelines. PubMed, EMBASE, Scopus, and Cochrane Library were searched from inception to March 2025 to identify eligible studies. Observational and interventional studies that reported the impact of health education on any health-related outcome among those attending a mass gathering were considered. A narrative synthesis of review results was performed to gather evidence. Recommendations were framed in the context of this evidence. **Results**: Of the 1731 records, only 17 studies met the inclusion criteria. These included cross-sectional (*n* = 10), pre-post design (*n* = 3), quasi-experimental (*n* = 2), randomized controlled trial (*n* = 1), and ethnographic (*n* = 1) studies. These studies involved participants attending hajj, umrah, and basketball events. The current evidence on health education in mass gatherings is highly varied in its objectives, intervention strategy, educational plan, mode of delivery, design, and reported outcomes. Most studies agreed that health education should be initiated by the country of origin and continued throughout the event. It is recommended that this education should be tailored to patient needs based on age, medical condition, and other personal factors, and given in the local language for better acceptability. Such sources can be provided in various forms, either online or offline, as per the participant’s convenience. **Conclusions**: The current evidence on the effectiveness of health education during mass gatherings, particularly in pilgrimage settings, is varied and inconsistent. Participant-tailored health education should be provided, preferably in the local language, through convenient formats.

## 1. Introduction

Events grounded in cultural, religious, social, or recreational traditions are integral to human society and are often classified as mass gatherings. The World Health Organization (WHO) defines mass gatherings as “an organized or unplanned event where the number of people attending is sufficient to strain the planning and response resources of the community, state, or nation hosting the event” [1]. Similarly, the Center for Disease Control and Prevention (CDC) Yellow Book describes it as “large numbers of people (>1000) at a specific location, for a specific purpose” [2]. Practically speaking, a mass gathering can be any assembly of people large enough to strain local resources [3].

Despite their positive societal and cultural value, mass gatherings pose a significant public health challenge due to the potential for infectious disease outbreaks and other health-related complications [4]. The convergence of large populations in confined or shared spaces during mass gatherings significantly increases health hazards. Outbreaks of respiratory infections, foodborne illnesses, heat-related illnesses, injuries, and exacerbation of non-communicable diseases have all been reported during such events [5,6]. Mass gatherings pose a significant infectious disease risk, with 72 respiratory outbreaks linked to such events from 2005–2014, with over 840,000 respiratory infection cases tied to the Union of European Football Associations (UEFA) Euro 2020 games [7], and recent measles surges in South Korea linked to international events [8]. These trends highlight the urgent need for targeted public health interventions and education. These challenges are further exaggerated by inadequate sanitation, poor crowd control, limited access to healthcare services, and the presence of high-risk groups such as the elderly or immunocompromised individuals [9,10]. To mitigate these risks, several preventive and public health strategies have been recommended, including the promotion of hand hygiene, mask wearing, environmental sanitation, vaccination, and crowd health surveillance [11,12,13,14].

Health education has emerged as a particularly important and cost-effective measure [11,14]. It plays a pivotal role in empowering individuals with the knowledge and motivation needed to adopt safer health practices, adhere to guidelines, and respond effectively during emergencies [15]. Health education influences health behavior, and its impact can be understood through various frameworks, one of which is the health belief model (HBM). The HBM posits that individuals’ engagement in health-related behaviors is determined by their perceptions of susceptibility, severity, benefits, and barriers, along with cues to action and self-efficacy [16,17,18]. Focusing on health education and health-related behaviors is especially important for mass gatherings due to the increased risk of infectious disease transmission in crowded, diverse settings [19]. In the context of mass gatherings, health-related behavior can be considered the first line of defense against infectious disease, particularly when medical interventions are limited [17]. Applying behavioral models like the HBM helps tailor messages so that they include psychological factors, such as perceived risk and self-efficacy, making interventions more effective [16,18]. Tailoring education to these psychological constructs increases the likelihood of meaningful behaviour change. Applying such a model ensures that interventions are not only informative but also motivational and behaviorally grounded [19,20], particularly at mass gatherings.

Although health education is widely recognized as a cornerstone of disease prevention, there is limited consolidated evidence evaluating its effectiveness in the specific context of mass gathering events. Most available studies and existing systematic reviews focus on broader intervention strategies, with health education often being included as part of a larger public health package rather than as an isolated component [11,14,21]. This lack of focused analysis limits our understanding of the standalone effectiveness of health education, particularly in the unique context of mass gatherings. To date, there is no comprehensive literature evidence that isolates and evaluates the specific contributions of health education in these settings. This scoping review addresses that gap by systematically mapping the available evidence on health education as an independent preventive measure at mass gatherings, offering novel insights into its role, implementation, and impact. Scoping reviews are particularly valuable for mapping the breadth and depth of existing literature on a topic, especially when the area is complex or not well defined. They help identify research gaps, clarify key concepts, and inform future studies or policy decisions by synthesizing diverse sources of evidence [22,23].

Given the increasing scale and frequency of mass gatherings, increased infections and antibiotic resistance worldwide and the health challenges they pose, it is essential to critically examine the available evidence regarding the role of health education. This information will help us planning the future events and prevention of mass gathering-associated health hazards. Therefore, this scoping review aims to explore and map the existing literature on the effectiveness of health education during mass gatherings, thereby identifying research gaps and guiding future public health policies and interventions.

## 2. Materials and Methods

This scoping review is reported according to the Preferred Reporting Items for Systematic Reviews and Meta-Analyses extension for Scoping Reviews (PRISMA-ScR) Guidelines [24]. The review was conducted following the Arksey and O’Malley’s scoping review framework which outlines five key stages in its process, with an optional sixth stage: (1) research question identification, (2) relevant study identification, (3) study selection, (4) data charting, (5) results reporting, and (6) consultation of an expert [25]. The protocol for this study is registered in PROSPERO with a registration number: CRD42025637008.

### 2.1. Stage 1: Identifying the Research Question

The research question was identified following a thorough preliminary literature review, which in turn emphasized the lack of evidence or consensus on the impact of health education among participants attending a mass gathering. The research question was clearly defined with the concept of interest (impact of health education on health outcomes), target population (participants attending any mass gathering), and context (any setting irrespective of purpose, geographical area, and type of participants).

### 2.2. Stage 2: Identifying Relevant Studies

A comprehensive search of the literature was conducted in PubMed, EMBASE, Scopus, and Cochrane library databases from inception to March 2025 to locate all relevant studies. The search terms entered were selected from Medical Subject Heading (MeSH) terms and the existing literature. The keywords identified were “mass gathering”, “mass event”, “crowd”, “hajj”, AND “health education”, “medical education”, “health promotion”. We also manually searched the bibliographies of relevant reviews to identify additional relevant studies. Detailed search strategy used in various databases is provided in Supplementary File S1.

### 2.3. Stage 3: Study Selection

This review employed Joanna Briggs Institute’s (JBI) framework for scoping reviews to match the study selection with the review question [26]. We included studies that met the predefined eligibility criteria.

### 2.4. Study Eligibility Criteria

#### 2.4.1. Population

The study population included any adult patients (aged > 18 years) attending any kind of mass gathering irrespective of their gender and ethnicity. We did not apply any other restriction to the population.

#### 2.4.2. Concept

The concept for this review was focused on health education in any of its forms provided to those attending a mass gathering. We considered all types of health education strategies that can be delivered through any medium of communication, such as online, offline, paper, videos, or a combination, through any method of delivery, such as in-person, virtual, smartphone, computer, or a combination. Any health-related outcomes, including knowledge, attitude, and practice, resulting from receiving health education were considered an outcome of interest. Studies that focused on basic education or knowledge without discussing health education were excluded. However, we did not consider studies that reported outcomes from those who provide the services, instead of those attending the mass gathering.

#### 2.4.3. Context

We considered any settings where a mass gathering can occur, including but not limited to religious events, sports, festivals, concerts, school events, and university events. We excluded those studies which involved individual enrolment without a mass gathering. Further, we did not consider interventions that were based on personal rather than preventive measures during mass gatherings.

#### 2.4.4. Type of Studies

We included all types of observational and interventional studies. Only studies published in the English language were considered for this review. Narrative or systematic reviews, conference abstracts, unpublished studies, and letters to the editor were excluded from this review. Qualitative studies were also excluded as our primary focus was on assessing the effectiveness and implementation of health education interventions, which are more directly addressed through quantitative and mixed-methods research.

Following the database search and removal of duplicates, the remaining studies were screened for titles and abstracts, and the full texts were evaluated for relevant citations using predefined inclusion criteria by two independent reviewers (A.A.A., and L.A.A.) from the research team. Disagreement was resolved through discussion or, when necessary, by a third independent evaluator (A.M.A.A. or A.A.K.).

### 2.5. Stage 4: Charting the Data

A pre-designed standardised data extraction form created in Microsoft Excel was used to avoid bias in the data abstraction process. The data were extracted by two independent authors (A.A.A., and L.A.A.) after verifying the data in the extraction form for its correctness and consistency. The collected data includes first author’s last name, country, year of publication, type of mass gathering, characteristics of participants, type of education, mode of education, and the outcomes. To ensure inter-reviewer reliability, data extraction was conducted independently by two reviewers using a standardized form. Any discrepancies were resolved through discussion and consensus, with a third reviewer (A.M.A.A. or A.A.K.) consulted when needed. This process helped minimize bias and enhance the consistency and accuracy of the extracted data.

### 2.6. Stage 5: Collating, Summarising, and Reporting the Results

Each eligible study was scrutinized for impacts of health education as a whole or as part of a preventive strategy and benefits among the patients attending a mass gathering. We considered studies that reported any health-related outcome. Studies lacking relevant information related to the research topic were excluded. All evidence extracted using this systematic process was summarized narratively and presented in a tabular form according to the health education strategy and its impact on health among participants who attended the mass gathering. Unfortunately, the high heterogeneity in study objectives, intervention adopted, mode of delivery, designs, and reported outcomes among the studies made it difficult to perform a statistical analysis, and it forced us to a narrative synthesis. Broadly, the outcomes were classified under the umbrella of improvement in knowledge, attitude, and practice and then detailed as per the individual outcomes. The available effect measures (such as percentage [%], mean difference [MD], or odds ratio [OR]) were summarized with a 95% confidence interval [CI] and *p*-value for significance.

## 3. Results

### 3.1. This Study Selection Process

**Figure 1:**
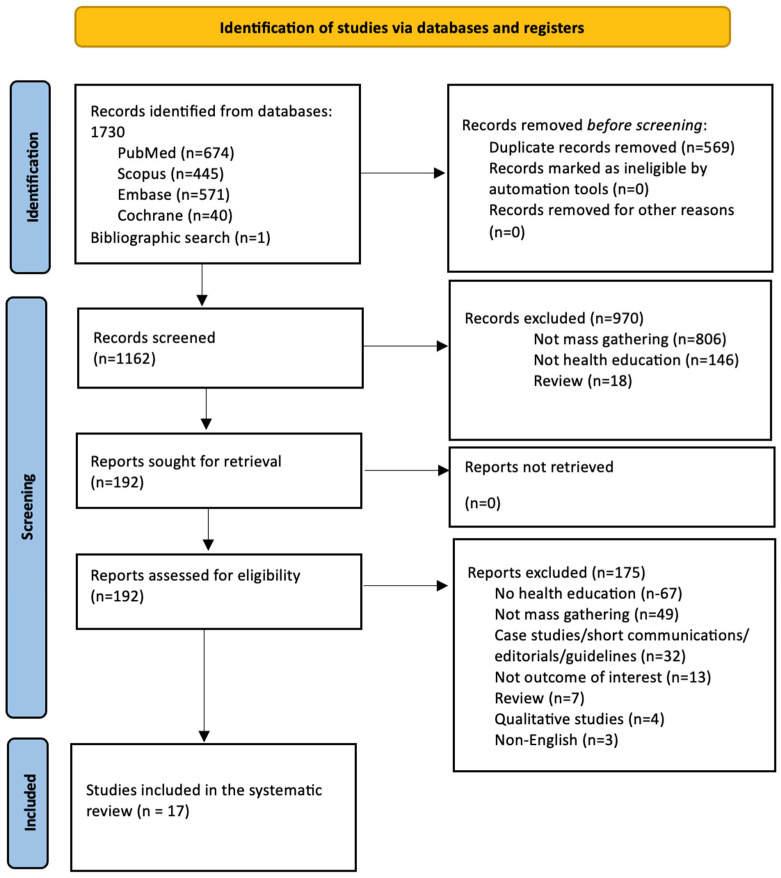
PRISMA flow diagram for study selection.

The literature search yielded a total of 1730 articles and one additional study from bibliographic search. After removal of duplicates (*n* = 569) and exclusion of 970 non-relevant articles during the initial screening, 192 relevant articles were considered for full-text screening. Of these, 175 studies were excluded for appropriate reasons. Finally, only 17 studies [27,28,29,30,31,32,33,34,35,36,37,38,39,40,41,42,43] were considered for this review. The PRISMA flow diagram of the study selection process is provided in Figure 1. The detailed reasons for exclusion of individual studies are provided in Supplementary File S2.

### 3.2. Characteristics of Included Studies

**Figure 2:**
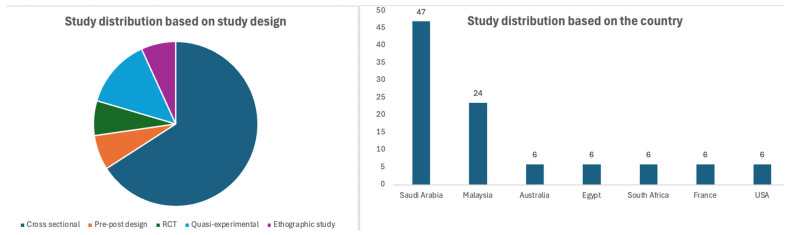
Study distribution based on study design and country.

**Figure 3:**
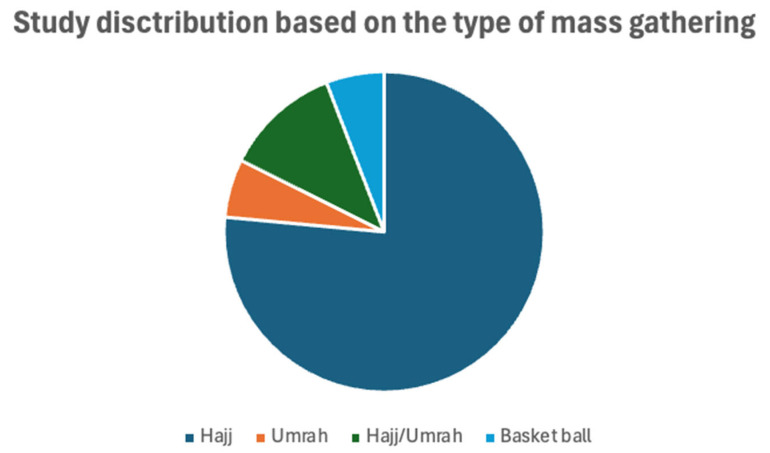
Study distribution based on the type of mass gathering.

Among the 17 included studies, 10 (58%) were cross-sectional studies. In the remaining studies, 3 (18%) were pre-post design, 1 (6%) was a randomized controlled trial (RCT), 2 (12%) were quasi-experimental, and 1 (6%) was an ethnographic study. All of these studies were published between 2008 and 2023. The country-wise distribution found that most studies were from Saudi Arabia (*n* = 8), followed by Malaysia (*n* = 4). The sample size varied from 57 to 1221 in the included studies. The studies included participants who attended hajj (*n* = 13), umrah (*n* = 1), hajj/umrah (*n* = 2), and basketball games (*n* = 1). Detailed information on the included studies is provided in Table 1. The study distribution, based on the study design, country, and the type of mass gathering, is presented in Figure 2 and Figure 3.

### 3.3. Characteristics of Health Education

Health education was either provided before or during the mass gathering, with a majority provided before the mass gathering event. This education was provided as a whole program or part of other education or general advice before the participants attended the mass gathering. These educational programs are targeted at improving the practice of better health etiquette during mass gatherings. The educational intervention was delivered or participants received the information in various methods such as in-person classes/workshops, videos/films, leaflets, magazines, television (TV) and radio programs, mobile applications, specialist travel clinics, specific hajj/ministry websites, other general websites, press and publications, social media, leaflets, medication labels, internet sources, and previous experiences. This education was provided by general practitioners, nurses, family and friends, any health care providers, selected physicians, pharmacists, health educators, and tour groups/hajj agents. The detailed information on health education is provided in Table 1.

### 3.4. Details and Focus of the Health Education

Although a good number of studies provided detailed information on the focus and contents of health education, some studies did not [28,29,30,41]. The focus of health education was asthma education [27], medication storage and handling [31], insulin storage [35], hand rubbing and mask use [32], prevention of influenza-like-illness [33], prevention of respiratory tract infection [36] medical seeking training [34], Middle East respiratory syndrome coronavirus-2 (MERS Cov-2) [39], cardio-pulmonary resuscitation (CPR) training [40], injury prevention [44] and general or various health education as part of the hajj/umrah pilgrimage [37,38,42,45] based on mass gatherings. Detailed information on various health education given to those who attend a mas gathering is provided in Table 2 and Supplementary File S3.

### 3.5. Effectiveness of Health Education

#### 3.5.1. Knowledge, Attitude, and Practice

A total of six studies explored the impact of health education on the knowledge, attitude, or practice of good health etiquette among individuals participating in mass gatherings. A study by Tobaiqy et al. [30] observed significantly higher healthy practices, such as use of face mask (*p* = 0.04), avoiding sun exposure (*p* = 0.03), and healthy practice scores (*p* = 0.02) among those who received health education. Interestingly, Turkestani et al.’s study [42] noted that health education provided to pilgrims in their mother tongue through the pictorial chart as well as the distribution of pictorial pamphlets can significantly increase their knowledge on healthy practices (*p* < 0.05). This study reported a better knowledge score after the intervention. However, three studies [32,36,37] recorded no significant effect of health education on participants’ health-related knowledge, attitudes, or practices. The study by Alamry et al. [43] reported that there was a good average practice score (6.7 ± 2.1 out of 8) among the participants.

#### 3.5.2. Vaccination

Three studies [28,29,41] reported that health recommendations and education could significantly improve the vaccine uptake. The study by Alqahtani et al. indicates that the advice from general practitioners (OR: 1.9) and the group leaders (OR: 2.1) almost doubled the vaccine intake compared to those who did not receive the education.

#### 3.5.3. Compliance with the Use of Face Mask

A quasi-experimental study by Goni et al. [33] reported that a smartphone-based health education intervention significantly increased the compliance on overall face mask use (25% vs. 2%; *p* < 0.001), use of N95 mask (21.2% vs. 2%; *p* = 0.005); disposing the masks (44.2% vs. 16%; *p* = 0.004); mask use in masjid (44.2% vs. 22%; *p* = 0.05); and mask use in crowded areas (32.7% vs. 14%; 0.026). Similar findings were reported in two additional studies conducted in Egypt and Saudi Arabia, both of which demonstrated a positive impact of health education on significantly (*p* < 0.05) improving face mask compliance among pilgrims (29, 30). The study by Alamry et al. [43] reports that 99.9% of participants used a mask in crowded places.

#### 3.5.4. Hand Hygiene

A study by Khamis et al. [29] reported that those who received a health education had better hand hygiene practice (58.7% vs. 11.8%) compared to those who did not. In contrast, a study by Salmuna et al. [32] reported no significant difference between pre- and post-hand-rub compliance (0.369) among hajj pilgrims in the intervention group.

#### 3.5.5. Respiratory Infections

A quasi-experimental study by Goni et al. [33] reported that a smartphone-based health education intervention significantly reduced the occurrence of symptoms of respiratory tract infections (9.6% vs. 26.0%; *p* = 0.038).

#### 3.5.6. Viral Infections

The study by Migault et al. [39] recorded that information about MERS-CoV provided by a nurse using an information leaflet increased the overall rate of correct responses (11 of 13) for MERS-CoV. However, the individual response to specific domains such as routes of transmission, symptoms, preventive behaviors to adopt, vaccines, and specific treatments remained lower than 50%.

#### 3.5.7. Asthma Education

A study by Ramli et al. [27] reported the provision of general advice regarding asthma management as part of hajj-training. However, there was very little or no intake of this education as it was optional for the participants. There was also a lack of an individualized educational plan in this study.

#### 3.5.8. Medication Storage

Receiving health education on medication storage was independently associated with good knowledge on appropriate storage of all medications (OR: 2.7; 95% CI: 1.4–5.0; *p* = 0.001) in hajj pilgrims [31]. Similar results were found among diabetic hajj pilgrims regarding insulin storage (0.52 ± 0.21 vs. 0.38 ± 0.19; *p* = 0.001) [35].

#### 3.5.9. Cardio-Pulmonary Resuscitation (CPR)

The study by Beskind et al. [40] recorded that CPR training was not significantly effective in improving participant responsiveness. However, a significant improvement was found in chest compression depth and hands-off time following the video intervention.

#### 3.5.10. Acceptance or Helpfulness of Health Education

Three studies indicated that the acceptance or helpfulness of health education among the participants was very high. The study by Tobaiqy et al. reported that 65% of the participants found the health education easier to follow and helpful, and 23% reported that the health education was beneficial to some extent [30]. Another study by Salmuna et al. recorded that there was a significant (*p* < 0.013) difference between pre-and post-hajj perception among hajj pilgrims in the intervention group [32]. Alamry et al. [43] reported that all of their participants benefited from the health education.

### 3.6. Practice Implications

- Start health education early and in the native language: Health education should begin in pilgrims’ home countries, using their native language, and continue in the host country (e.g., Saudi Arabia during Hajj/Umrah) [27,29,30,31,34,35,42].
- Build a strong educator–participant relationship: Establishing trust and communication between health educators and pilgrims is key to effective education and care delivery during mass gatherings [31,46].
- Tailor education to pilgrims’ needs: Education should consider individual factors such as health literacy, age, medical conditions, and preferences (e.g., using visuals or simplified language) [28,30,31,38].
- Boost participation through incentives and requirements: Developing engaging methods and possibly making educational sessions mandatory could improve participation and information uptake [28,30,38].
- Focus on preventive measures: Educational materials should emphasize preventive health practices alongside general advice to maximize impact [33,36,45].
- Use technology to increase reach and engagement: Mobile apps and social media platforms can make health education more accessible and widely accepted [33,36].
- Develop standardized content in collaboration with authorities: A standard health education package should be created with the Ministry of Health to ensure consistency. It could be shared via official travel websites and transportation systems (e.g., flights, buses, ships) [42].

### 3.7. Research Implications

- Identify system-level barriers: More research is needed to understand the system level challenges in implementing health education programs for mass gatherings [44]. Identifying the type of gathering is very important to understand these barriers [47].
- Conduct large, long-term comparative studies: There is a need for large-scale studies comparing the effectiveness of different education methods, especially in terms of long-term knowledge retention [44].
- Examine the impact of information sources and relationships: Studies should explore how various information channels and the provider–receiver dynamic influence health education outcomes [27,30].
- Define what effective health education looks like: More research should clarify what constitutes effective health education for pilgrims. This should cover content, format, access, delivery, provider, language, and follow-up strategies [30].
- Understand pilgrims’ perceptions and knowledge gaps: Investigating pilgrims’ views will help identify knowledge gaps, which can inform the design of more targeted and effective education programs [32,48]. There is a lack of research evidence on how pilgrims perceive risks and adopt information, and how best to interact with their willingness to be trained in preventive measures [13].

## 4. Discussion

### 4.1. General Background of Discussion

Mass gatherings are increasing globally due to factors such as religious pilgrimages, international sporting events, and festivals. This rise presents both opportunities for cultural exchange and challenges in managing public health, safety, and logistics [2,49]. Health education is an important factor that improves practice and decreases disease burden during these events. However, this has rarely been addressed in the literature. This scoping review aimed to explore the existing knowledge on the impact of health education among mass gathering attendees. It also identified gaps in the current literature and provided practical and research implications to guide future efforts by stakeholders and researchers.

### 4.2. Findings from the Evidence and Discussion

Available evidence on health education for mass gatherings is scarce or not provided in an adequate manner. The need for structured and culturally sensitive health education aligns with global frameworks, such as the WHO guidelines on mass gatherings. These should emphasize risk communication, health promotion, and community engagement as core pillars of preparedness and response strategies [1,50]. Moreover, this approach supports Sustainable Development Goal 3 that focuses on good health and well-being, by promoting health literacy and access to essential health information, thereby empowering individuals to make informed decisions and reduce preventable health risks during mass gatherings [51]. Tailored health education aligned with these goals not only addresses individual behavior change but also contributes to broader public health resilience in line with global health objectives.

Our current review summarized beneficial effects of health education on improving the knowledge, attitude, and practice of pilgrims. However, these were based on the type and contents of the education provided. Furthermore, the language in which such education is delivered and who provides this education matters greatly in the uptake among the pilgrims [28,38,41,42]. Some pilgrims were observed to possess good health attitudes and practices, although their education level was low [46]. This might be due to their experience or personal knowledge from a previous pilgrimage. Interestingly, all studies reported that health recommendations and education could significantly improve the vaccine uptake, especially when advised by general practitioners or group leaders [28,29,41]. This evidence indicates that health education for pilgrims is highly effective when tailored in content, language, and delivered by trusted sources. Even those with lower education levels can exhibit good health practices, likely due to prior experiences. Trusted figures like general practitioners and group leaders play a key role in boosting vaccine uptake.

The mode of delivery plays a very important role in participant education, especially when there is room for continuous education [52]. This can be possible through digital delivery or mobile/online-based education where the participants can have continuous access. Additionally, this also can ensure access for their healthcare providers when needed [53]. Current challenges, such as digital literacy, participant convenience, internet availability, and availability of health educators, should be considered for effective digital health delivery [54]. Additionally, converting these education materials into alternative forms like short videos, documentaries, and audio-visuals with original case scenarios will help participants recollect this information in a stronger way and has been found to be most effective [53,55]. Hence, regional health authorities should encourage these initiatives for better health practices among those attending a mass gathering.

Evidence from this review indicates that health education can significantly increase compliance with regular face mask use in various settings like the masjid or crowded areas. Additionally, it improves rates of safe disposal and handling of masks [29,30,33]. Although health education significantly improved hand hygiene practices [29], compliance with hand-rubbing was not improved [32]. This suggests a gap between knowledge acquisition and behavioral execution in specific practices [12,56,57]. A recent pilot clinical trial explored the relationship between practicing hand hygiene and rates of ARI in Umrah pilgrimage, and no significant relation could be established [58]. However, this study established the feasibility of conducting large clinical trials in this domain. Factors such as accessibility, cultural preferences, or misconceptions about hand-rub effectiveness may contribute to this discrepancy [37,59]. Therefore, targeted interventions addressing these specific barriers, alongside general education, may be necessary to ensure consistent adoption of all recommended hygiene practices during mass gatherings.

Health education can significantly decrease the occurrence of respiratory infections [33], and increase the overall knowledge on these infections [39]. However, domain-specific knowledge on routes of transmission, symptoms, preventive behaviors to adopt, vaccines, and specific treatments remained lower than 50% among participants [39]. While general health education has shown a positive impact on reducing respiratory infections and improving overall awareness, there is a limited understanding of specific aspects, such as transmission routes, symptom recognition, nonpharmaceutical interventions, personal protective behaviors and environmental hygiene, as well as targeted prevention strategies such as hygiene promotion, mask use, and social distancing [60,61]. This highlights a critical gap in educational outreach. Although one recent trial failed to establish a relationship between the best hand-hygienic practice and incidence of respiratory infection, it could open the possibilities to conducting large trials in this domain [58]. It is important to note that there was no or little uptake of asthma education among a set of hajj-pilgrimage attendees [27]. However, it was provided as optional education along with other general advice. This indicates that broad messages alone may not be sufficient; instead, more focused, topic-specific content is needed to deepen understanding [62]. The low uptake of asthma education among certain pilgrim groups further emphasizes the need for tailored interventions that address individual health conditions and prioritize high-risk populations. Strengthening the depth and reach of education programs could significantly enhance preparedness and health outcomes during mass gatherings [11,28,30,31,38].

Health education significantly improved the knowledge and practice of medication storage among all pilgrims and those with specific diseases like diabetes [31,35]. The improvement in medication storage practices among pilgrims reflects the potential of targeted health education to influence practical, disease-specific behaviours [35,63]. Proper medication management is particularly crucial in extreme environments like those encountered during mass gatherings, where temperature control and accessibility can be challenging. For individuals with chronic conditions such as diabetes, safe storage directly impacts treatment effectiveness and health outcomes [64,65,66]. This suggests that when health education addresses both general and condition-specific needs, it can empower pilgrims to take proactive steps in managing their health more effectively throughout their journey.

There is evidence that indicates musculoskeletal benefits through the application of personalized intervention, especially when more physical activities are involved in events or gatherings [44]. It is very important to provide specific tailored musculoskeletal health-based interventions for music or band students, for example, and those preparing for or performing in concerts, given the physical demands required [67,68,69]. Repetitive movements, prolonged practice sessions, and performance-related stress can lead to muscular strain and pain over time. The effectiveness of a short, structured injury prevention session highlights the value of integrating such strategies into music education programs, especially in universities [69,70]. By equipping students with practical tools to protect their physical health, institutions can help sustain their long-term performance ability and overall well-being.

No significant benefit was observed with CPR education or health education in reducing the outpatient and emergency room visits among the pilgrims [40,45]. However, a significant improvement was found in hands-off time during chest compressions following the video intervention. This might be due to participants’ inability to grasp this information and the need for more tailored information to be adapted in the future. Though none of the studies addressed the impact of education in preventing hand-cut injuries while slaughtering, it is very important to educate pilgrims on safe and preventive measures to take while performing the slaughtering [14,71].

### 4.3. Limitations and Gaps

Though this scoping review extensively addressed the current knowledge and understanding on the impact of health education in mass gatherings, there are gaps and limitations in the existing evidence. The lack of organized education programs and lesser intake of these programs [27,29,32,46], self-reported outcomes in studies due to cross-sectional study nature and associated recall bias [31,33,35,36,38,45,48,60], short duration, lesser sample size, and lack of follow-up [33,34,40,44,45] were limitations of this review. Additionally, high heterogeneity in health education and related outcomes might have hampered the complete generalizability of findings. Finally, the exclusion of non-English language studies, grey literatures, and qualitative studies might have contributed to us missing some important papers. Additionally, hajj and umrah are the largest mass gathering across the globe and there is a possibility that many papers have been published in regional languages. However, a comprehensive search strategy and use of multiple databases may have helped us to locate the maximum possible literature on this topic.

## 5. Conclusions

Overall, the evidence from this review underscores the vital role of targeted and well-delivered health education in improving health-related knowledge, attitudes, and practices among individuals participating in mass gatherings. Brief interventions which focus on targeted participants can yield meaningful benefits. From enhancing preventive behaviors to improving medication storage and reducing infections, health education proves to be a powerful tool in preparing for mass gathering events. However, the current lack of conclusive evidence highlights the need for further research to strengthen and expand this area of study. Trusted sources and culturally tailored content significantly influence effectiveness, and even brief interventions can yield meaningful benefits, as seen in injury prevention among music students. There was an absolute lack of evidence in assessing the effectiveness of health education on clinical outcomes such as morbidity, mortality, and quality of life. Future efforts should focus on refining educational strategies to ensure they are accessible, relevant, and responsive to the unique needs of diverse populations in high-risk, high-density settings.

### Deviation from the Registered Protocol

Though the actual protocol for this study was registered to understand the effectiveness of health education in reducing morbidity and mortality during mass gathering events (PROSPERO ID: CRD42025637008), we could not find any studies on it. Hence, we modified our outcomes into “all health-related outcomes” instead of “morbidity and mortality”.

## Figures and Tables

**Table 1:** The characteristics of included studies and health education.

Author, Year	Country	Study Design	Type of Participants	Type of Health Education	No. of Participants	Pattern/Sources of Health Education
Ramli R, 2022 [27]	Malaysia	Ethnographic study	Hajj pilgrims	Optional asthma education as part of health examination conducted in 11 public and 2 primary clinics before hajj departure	NR	16 educational sessions, including 1 on health
Alqahtani AS 2016 [28]	Australia	Cross-sections study	Hajj pilgrims	Pre-travel professional travel health advice	236	General practitioners (51%); specialist travel clinic (15%), specific hajj website (8%); ‘Smartraveller’ website (7%); not received (33%)
Khamis NK [28]	Egypt	Cross-sectional study	Hajj pilgrims	Pre-travel health education	248	34.4% received health education
Tobaiqy M 2020 [30]	Saudi Arabia	Cross-sectional survey	Umrah pilgrims	Pre-travel health education	1012	Press and publications (9.7%); family and friends (12.5%); lectures (25.8%); social media (5%); travel clinics (13.2%); health care providers (12%); Saudi MoH website (1.6%); other websites (5.1%); other sources (18%)
Yezli S 2021 [31]	Saudi Arabia	Cross-sectional survey	Hajj pilgrims	Health education on medication handling and Storage	1221	Physicians (73.7%); pharmacists (39.4%); medication label itself (28.2%), internet and family members (6.6%)
Salmuna ZN 2019 [32]	Malaysia	Open label RCT	Hajj pilgrims	Health education on hand-hygiene	500 (Int: 250; Cont: 250)	One-to-one education
Goni MD 2023 [33]	Malaysia	Quasi-experimental study	Hajj/Umrah pilgrims	Smartphone-based health education intervention guided by the Health Belief Model on prevention of influenza-like illnesses	102	Smartphone application
Mushi A 2021 [34]	South Africa	Cross-sectional survey	Hajj pilgrims	Pre-hajj training and health promotion	1138	In-person
Yezli S 2021 (2) [35]	Saudi Arabia	Cross-sectional survey	Diabetic hajj pilgrims	Education on insulin storage and handling	227	Any (83.6%); physician (77.8%); pharmacist (59.6%); label (5.3%); internet (8.2%); other (7.6%)
Goni MD 2021 [36]	Malaysia	Quasi experimental	Hajj/Umrah pilgrims	Smartphone-based health education intervention guided by the Health Belief Model on prevention of respiratory diseases	130	Smartphone application
Mahdi H 2020 [37]	Saudi Arabia	Cross-sectional survey	Hajj pilgrims	Pre-hajj health advice	348	Any health advice (75.6%); Doctors (11.1%); Special Hajj websites (18.8%); Tour groups (18.5%); Family and friends (30.1%); General websites (21.5%); and MoH recommendations (66.4%)
Alqahtani AS 2019 [38]	Saudi Arabia	Cross-sectional survey	Hajj pilgrims	Pretravel health-advice-seeking behavior	344	Any (44%); media sources (27.6%); travel clinic (14.5%); family doctor/general practitioner (9.3%); MoH (3.7%); non-medical sources (16.8%); internet sources (7.8%); family and friends (6.1%); and hajj travel (2.9%)
Migault C 2019 [39]	France	Cross-sectional study	Hajj pilgrims	Pre-hajj education health program about Middle East respiratory syndrome	82	In-person
Beskind LD 2017 [40]	USA	Pre-post interventional	Lay bystanders attending the basketball games	UBV	96 (Pre-intervention: 45, Post intervention: 51)	Video education
Barasheed O 2013 [41]	Saudi Arabia (Australians)	Cross-sectional survey	Hajj pilgrims	Pre-Hajj advice for vaccination	995 (2011: 442, 2012: 553)	In person (Tour groups)
Turkestani A 2013 [42]	Saudi Arabia	Pre-post intervention	Hajj pilgrims	Health education	300	In person (health educators)
Alamri FA et al., 2018 [43]	Saudi Arabia	Post-intervention	Hajj pilgrims	Health education	4925	In-person (medical staff including 163 doctors and 1463 technicians)

MoH: Ministry of Health; NR: not reported; RCT: randomized controlled trial; UBV: ultra-brief CCO-CPR video.

**Table 2:** Details on Health education and outcomes.

Author, Year	Contents of Health Education	Type of Outcomes	Effectiveness Outcomes	Implications	Limitations
Ramli R, 2022 [27]	General advice (to bring the medications, check expiry, diet control, and exercise)	Knowledge	There was little/no individualized asthma education	Doctor-participant relation is very important; optimal management assessment by physician; provision of printed or electronic educational resources	Lack of organized education for pilgrims
Alqahtani AS 2016 [28]	NR	Experience and practice	Positive experience with the advice (53.6%), negative experience (19%). Increased vaccination among those who had advice from GP (OR:1.9); group leader (OR:2.1)	Awareness, especially to elderly adults and those with pre-existing illness, would be highly beneficial	NR
Khamis NK [28]	NR	Practice	Pilgrims who received health education before hajj conducted lower risky behaviors and increased vaccination (87.5% vs. 66.7%), use of protective face mask (24% vs. 3.5%), and hand hygiene (58.7% vs. 11.8%) compared to others	Intensified health education campaigns should be conducted for all pilgrims in their mother countries and Saudi Arabia. This education should contain information on hajj-related health behaviors and how to avoid the conduct of poor behaviors.	Only a lower proportion of participants received the health education
Tobaiqy M 2020 [30]	NR	Practice	The use of face mask (*p* = 0.04), avoiding sun exposure (*p* = 0.03), and healthy practice score (*p* = 0.02) was significantly higher among those who had any form of health education compared to those who did not	Health education lectures are the most adapted strategy The content and type of information to include targeting the pilgrim’s language, literacy, and economic aspects. Future studies should focus on the development of accessible health education content in a form that engages pilgrims from diverse backgrounds to promote comprehensive preventative measures during mass religious gatherings and pilgrimages	NR
Yezli S 2021 [31]	General advice (Medication Handling and Storage)	Knowledge and practice	Receiving health education on mediation storage was independently associated with good knowledge (OR: 2.7; 95% CI: 1.4–5.0; *p* = 0.001) 4.7% reported not storing medications properly, and 7.6% would use medications that they know were stored inappropriately	Health education should start at the country of origin and continue during pilgrims’ stay in KSA, and should be led by physicians and pharmacists Beneficial to identify pilgrims with limited health literacy and offer them tailored medication counseling that fits their needs	No information on type and number of medications, self-reported outcome
Salmuna ZN 2019 [32]	One-to-one demonstration on how to use handrub provided as well as pamphlets on handrub usage, precautionary measures such as dietary habits, the correct way to use facemask and handrub	Knowledge, practice, and perception	There is no significant difference between pre- and post-hajj knowledge (*p* = 0.889), practice (*p* = 0.868), and hand-rub compliance (0.369) among hajj pilgrims in the intervention group. There is a significant (*p* < 0.013) different between pre- and post-hajj perception among hajj pilgrims in intervention group.	Understanding the perception would assist in pinpointing the knowledge gaps which may be utilized in developing educational programs in order to increase the awareness of the hajj pilgrims. Tailor the health education based on the age and level of education. Educational book with pictures rather than wordy components.	Inadequate awareness
Goni MD 2023 [33]	Hajj health educational module provided in pre-during-and post-hajj, followed by formative assessments on prevention of influenza-like illness developed in collaboration with private hajj companies	Practice	Health education significantly reduced the occurrence of RTI symptoms (9.6% vs. 26.0%; *p* = 0.038); and increased the compliance such as use of face mask (25% vs. 2%; <0.001); N95 mask (21.2% vs. 2%; *p* = 0.005); disposing the masks (44.2% vs. 16%; *p* = 0.004); mask use in masjid (44.2% vs. 22%; *p* = 0.05); and mask use in crowded areas (32.7% vs. 14%; 0.026).	The inclusion of preventive measures in health education is very important	Short period of enrollment and outcome assessment; Control group exposure to other sources of information; Use of self-reported outcomes
Mushi A 2021 [34]	Health promotion and training on how and where to seek medical information, health risks during hajj, and health messages on different practices	Practice	Decreased the risky practices	Health authorities in the countries of origin are encouraged to provide health education for pilgrims prior to arrival in KSA for Hajj.	Fewer participants enrollment
Yezli S 2021 (2) [35]	Education on insulin storage	Knowledge	Previous health education significantly improved their knowledge score on appropriate insulin storage (0.52 ± 0.21 vs. 0.38 ± 0.19; *p* = 0.001)	Health education for diabetic patients should start at the country of origin and continue during the Hajj pilgrimage, led by physicians and pharmacists, and the pilgrims’ medical missions	A smaller sample size, the Sampling methodology, and the potential for volunteer bias
Goni MD 2021 [36]	Smartphone-based module on knowledge, attitude, and practice regarding respiratory tract infection prevention	Knowledge, attitude, practice	There was no significant improvement in knowledge (17.46 vs. 16.15; *p* = 0.169), attitude (33.36 vs. 31.96; *p* = 0.101), and practice (28.10 vs. 26.58; *p* = 0.078) between intervention and control groups.	Mobile phone technology to gather information on infections associated with mass gathering and travelers makes compliance with prevention practices achievable.	Intrinsic deficiencies and hindrances in implementation.
Mahdi H 2020 [37]	General advice	Knowledge	No significant difference in practice based on the knowledge level	Foreign pilgrims are generally better informed and better prepared for Hajj travel since health authorities in their countries of origin are mandated to ensure health advice to pilgrims on communicable diseases Giving advice by Islamic scholars about the importance of alcohol-based hand rubs and reinforcing how this practice does not harm pilgrims could potentially eliminate taboos surrounding the use of alcohol-based hygienic products and, in turn, enhance compliance	Domestic pilgrims who did not have any travelling
Alqahtani AS 2019 [38]	General advice	Practice	Those who had chronic conditions were more likely to seek advice from medical sources than those who did not have any chronic conditions (adjusted odds ratio [aOR]: 2.6, 95% CI: 1.1–6.4, *p* = 0.03)	NR	Lack of causal inference, recall bias due to self-reported survey
Migault C 2019 [39]	Information about MERS-CoV provided by a nurse, using an information leaflet	Knowledge	Delivery of educational information increased the overall rate of correct responses (11 of 13) about MERS-CoV. However, the individual response to specific domains such as routes of transmission, symptoms, preventive behaviors to adopt, vaccines, and specific treatments remained lower than 50%	Information targeting the public is the preferred means to implement infection control	Though the level of knowledge is improved, not effective in controlling the epidemic
Beskind LD 2017 [40]	30 s UBV was shown on screen in the middle of the gymnasium (Jumbotron) that illustrated a step-by-step demonstration on how to perform CCO-CPR using the three C’s (Check, Call 911, Compress).	Practice	No significant difference between groups for the proportion of participants’ responsiveness (Call to 911/AED, started compressions within 2 min) and chest compression rate. Significant improvement found in chest compression depth hands-off time following the video intervention.	Mass media interventions to improve the performance of bystander CPR have grown and become a common method for public awareness campaigns	Study conducted in a simulated situation that may not be applicable to how participants would perform under the stress of a true emergent scenario, selection bias due to convenient sampling
Barasheed O 2013 [41]	NR	Practice	89% Australian pilgrims received influenza vaccine in 2012 due to tour group leaders’ recommendation, awareness about the availability of influenza vaccine, and an increased perception of risk	Recommendations of religious leaders like Imams and tour group leaders were important in enhancing the uptake of influenza vaccine among pilgrims (Enhancing the prevention strategies to RTIs).	Level of education and occupation could influence pilgrims’ perception about influenza vaccine, but these data were not collected
Turkestani A 2013 [42]	Provided effective health education to pilgrims in their mother tongue at their dormitories in the holy places through the pictorial chart as well as the distribution of pictorial pamphlets	Knowledge	Health education significantly increased the rate of correct answers (*p* < 0.05) 99.6% agreed the HEA program aboard the buses was beneficial	HEA program should continue during hajj season Research should be focused on understanding the impact of health education on any change in health Methods to provide standardized, pre-departure health education to pilgrims should be explored. Health education materials should be prepared in concert with the Ministry of Health and shared through working with air carriers and charter companies serving Hajj ports of entry to provide in-flight health education videos	NR
Alamri FA et al., 2018 [43]	Health education	Practice	All (99.6%) participants benefited from health education. The Mean and standard deviation of practice score were 6.7 ± 2.1 out of 8. Practice score in general was good. 99.9% of participants used masks in crowded places	Public awareness through social media should be used for education; gender is not a factor that affects health education	Older population had a high chance of comorbidities and health issues

AED: Automated External Defibrillator; CI: confidence interval; CPR: cardio-pulmonary resuscitation; HEA: health education ambassador; KSA: Kingdom of Saudi Arabia; OR: odds ratio; NR: not reported; RTI: respiratory tract infection; UBV: ultra-brief CCO-CPR video.

## Supplementary Materials

The following supporting information can be downloaded at: https://www.mdpi.com/article/10.3390/healthcare13151926/s1, Supplementary File S1: The search strategy in databases; Supplementary File S2: The list of excluded studies with reasons; Supplementary File S3: The detailed contents of health education.

## Abbreviations

WHO	World Health Organization
CDC	Center for Disease Control
PRISMA-ScR	Preferred Reporting Items for Systematic Reviews and Meta-Analyses extension for Scoping Reviews
JBI	Joanna Briggs Institute’s
RCT	randomized controlled trial
CPR	Cardio-pulmonary resuscitation

## References

[1] World Health Organization (2015). Public Health for Mass Gatherings: Key Considerations.

[2] Halsey E. (2025). CDC Yellow Book 2026: Health Information for International Travel.

[3] Al-Tawfiq J.A., Gautret P., Benkouiten S., Memish Z.A. (2016). Mass Gatherings and the Spread of Respiratory Infections. Lessons from the Hajj. Ann. Am. Thorac. Soc..

[4] David S., Roy N. (2016). Public health perspectives from the biggest human mass gathering on earth: Kumbh Mela, India. Int. J. Infect. Dis..

[5] Parker S., Steffen R., Rashid H., Cabada M.M., A Memish Z., Gautret P., Sokhna C., Sharma A., Shlim D.R., Leshem E. (2024). Sacred journeys and pilgrimages: Health risks associated with travels for religious purposes. J. Travel Med..

[6] Memish Z.A., Steffen R., White P., Dar O., Azhar E.I., Sharma A., Zumla A. (2019). Mass gatherings medicine: Public health issues arising from mass gathering religious and sporting events. Lancet.

[7] Dehning J., Mohr S.B., Contreras S., Dönges P., Iftekhar E.N., Schulz O., Bechtle P., Priesemann V. (2023). Impact of the Euro 2020 championship on the spread of COVID-19. Nat. Commun..

[8] Johnson D.R. (2024). Health Communications Matter: A Comparative Case Study of Best Practices to Combat Misinformation and Disinformation During the COVID-19 Pandemic. Ph.D. Thesis.

[9] Dawood H.N., Al-Jumaili A.H., Radhi A.H., Ikram D., Al-Jabban A. (2023). Emerging pneumococcal serotypes in Iraq: Scope for improved vaccine development. F1000Research.

[10] Adegboye O., Alele F., Pak A., Alakunle E., Emeto T., Leggat P., Okeke M. (2024). Monkeypox Outbreak 2022, from a Rare Disease to Global Health Emergence: Implications for Travellers. Adv. Exp. Med. Biol..

[11] Alhussaini N.W.Z., Elshaikh U.A.M., Hamad N.A., Nazzal M.A., Abuzayed M., Al-Jayyousi G.F. (2022). A scoping review of the risk factors and strategies followed for the prevention of COVID-19 and other infectious diseases during sports mass gatherings: Recommendations for future FIFA World Cups. Front. Public Health.

[12] Sun X., Keim M., He Y., Mahany M., Yuan Z. (2013). Reducing the risk of public health emergencies for the world’s largest mass gathering: 2010 World Exposition, Shanghai China. Disaster Health.

[13] Almehmadi M., Alqahtani J.S. (2023). Healthcare Research in Mass Religious Gatherings and Emergency Management: A Comprehensive Narrative Review. Healthcare.

[14] Ridda I., Mansoor S., Briggs R., Gishe J., Aatmn D., Laher I. (2019). Preparedness for Mass Gathering During Hajj and Umrah. Handbook of Healthcare in the Arab World.

[15] Hutton A., Ranse J., Munn M.B. (2018). Developing Public Health Initiatives through Understanding Motivations of the Audience at Mass-Gathering Events. Prehosp. Disaster Med..

[16] Champion V.L. (1984). Instrument development for health belief model constructs. Adv. Nurs. Sci..

[17] Katatsky M.E. (1977). The health belief model as a conceptual framework for explaining contraceptive compliance. Health Educ. Monogr..

[18] Jaccard J. (1975). A theoretical analysis of selected factors important to health education strategies. Health Educ. Monogr..

[19] Ricci L., Villegente J., Loyal D., Ayav C., Kivits J., Rat A. (2022). Tailored patient therapeutic educational interventions: A patient-centred communication model. Health Expect..

[20] Barrera M., Castro F.G., Strycker L.A., Toobert D.J. (2013). Cultural adaptations of behavioral health interventions: A progress report. J. Consult. Clin. Psychol..

[21] Ranse J., Hutton A., Keene T., Lenson S., Luther M., Bost N., Johnston A.N.B., Crilly J., Cannon M., Jones N. (2017). Health Service Impact from Mass Gatherings: A Systematic Literature Review. Prehosp. Disaster Med..

[22] Reghunath S.R., Rashid M., Chandran V.P., Thunga G., Shivashankar K., Acharya L.D. (2023). Factors contributing to the adverse drug reactions associated with the dipeptidyl peptidase-4 (DPP-4) inhibitors: A scoping review. Diabetes Metab. Syndr..

[23] Prathiksha A.S., Shantaram P.M., Rashid M., Poojari P.G., Nair S., Acharya L.D., Thunga G. (2024). Determinants of and barriers to diabetes care among patients with serious mental illness: A scoping review with recommendations. Diabetes Metab. Syndr..

[24] Tricco A.C., Lillie E., Zarin W., O’Brien K.K., Colquhoun H., Levac D., Moher D., Peters M.D.J., Horsley T., Weeks L. (2018). PRISMA Extension for Scoping Reviews (PRISMA-ScR): Checklist and Explanation. Ann. Intern. Med..

[25] Arksey H., O’malley L. (2005). Scoping studies: Towards a methodological framework. Int. J. Soc. Res. Methodol..

[26] Peters M.D., Godfrey C.M., Khalil H., McInerney P., Parker D., Soares C.B. (2015). Guidance for conducting systematic scoping reviews. JBI Evid. Implement..

[27] Ramli R., Hanafi N.S., Hussein N., Lee P.Y., Ghazali S.S., Cheong A.T., Abu Bakar A.I., Samad A.A., Abdullah S., Pinnock H. (2022). Hajj health examination for pilgrims with asthma in Malaysia: An ethnographic study. J. Glob. Health.

[28] Alqahtani A.S., Wiley K.E., Tashani M., Willaby H.W., Heywood A.E., BinDhim N.F., Booy R., Rashid H. (2016). Exploring barriers to and facilitators of preventive measures against infectious diseases among Australian Hajj pilgrims: Cross-sectional studies before and after Hajj. Int. J. Infect. Dis..

[29] Khamis N.K. (2008). Epidemiological pattern of diseases and risk behaviors of pilgrims attending mina hospitals, hajj 1427 h (2007 g). J. Egypt. Public Health Assoc..

[30] Tobaiqy M., Alhasan A.H., Shams M.M., Amer S.A., MacLure K., Alcattan M.F., Almudarra S.S. (2021). Assessment of Preventative Measures Practice among Umrah Pilgrims in Saudi Arabia, 1440H-2019. Int. J. Environ. Res. Public Health.

[31] Yezli S., Yassin Y., Mushi A., Balkhi B., Stergachis A., Khan A. (2021). Medication Handling and Storage among Pilgrims during the Hajj Mass Gathering. Healthcare.

[32] Salmuna Z., Hashim S., Hasan H., Abdul Aziz A., Nyi N.N., Mohamed Z., Harun A., Abdul Rahman Z. (2019). Knowledge, perceptions and practices of Malaysian hajj pilgrims for prevention of influenza-like illness (ILI) in 2013 hajj season. IIUM Med. J. Malays..

[33] Goni M.D., Hasan H., Naing N.N., Wan-Arfah N., Deris Z.Z., Arifin W.N., Baaba A.A. (2023). Impact of a Health Education Intervention on the Incidence of Influenza-Like Illnesses (ILI) During Hajj via Smartphone Application. J. Immigr. Minor. Health.

[34] Mushi A., Yassin Y., Khan A., Alotaibi B., Parker S., Mahomed O., Yezli S. (2021). A Longitudinal Study Regarding the Health Profile of the 2017 South African Hajj Pilgrims. Int. J. Environ. Res. Public Health.

[35] Yezli S., Yassin Y., Mushi A., Balkhi B., Khan A., Scaramuzza A. (2021). Insulin Knowledge, Handling, and Storage among Diabetic Pilgrims during the Hajj Mass Gathering. J. Diabetes Res..

[36] Goni M.D., Naing N.N., Hasan H., Wan-Arfah N., Deris Z.Z., Arifin W.N., Baaba A.A., Adam B.M., Arshad M.R. (2021). Effectiveness of a Novel Smartphone Health Education Intervention in Enhancing Knowledge, Attitudes, and Practices for the Prevention of Respiratory Tract Infections Among Private Hajj Pilgrims from Malaysia. Front. Public Health.

[37] Mahdi H., Alqahtani A., Barasheed O., Alemam A., Alhakami M., Gadah I., Alkediwi H., Alzahrani K., Fatani L., Dahlawi L. (2020). Hand Hygiene Knowledge and Practices among Domestic Hajj Pilgrims: Implications for Future Mass Gatherings Amidst COVID-19. Trop. Med. Infect. Dis..

[38] Alqahtani A.S., Althimiri N.A., BinDhim N.F. (2019). Saudi Hajj pilgrims’ preparation and uptake of health preventive measures during Hajj 2017. J. Infect. Public Health.

[39] Migault C., Kanagaratnam L., Hentzien M., Giltat A., Nguyen Y., Brunet A., Thibault M., Legall A., Drame M., Bani-Sadr F. (2019). Effectiveness of an education health programme about Middle East respiratory syndrome coronavirus tested during travel consultations. Public Health.

[40] Beskind D.L., Stolz U., Thiede R., Hoyer R., Robertson W., Brown J., Ludgate M., Tiutan T., Shane R., McMorrow D. (2017). Viewing an ultra-brief chest compression only video improves some measures of bystander CPR performance and responsiveness at a mass gathering event. Resuscitation.

[41] Barasheed O., Rashid H., Heron L., Ridda I., Haworth E., Nguyen-Van-Tam J., Dwyer D.E., Booy R., on behalf of the Hajj Research Team (2014). Influenza vaccination among Australian Hajj pilgrims: Uptake, attitudes, and barriers. J. Travel Med..

[42] Turkestani A., Balahmar M., Ibrahem A., Moqbel E., Memish Z. (2013). Using health educators to improve knowledge of healthy behaviour among Hajj 1432 (2011) pilgrims. East. Mediterr. Health J..

[43] Alamri F., Amer S., Alhraiwil N. (2018). Knowledge and practice after health education program among Hajj 1438 H (2017) Pilgrims. Saudi Arabia J. Epidemiol. Health Care.

[44] Wolff A.L., Ling D.I., Casey E.K., Toresdahl B.G., Gellhorn A.C. (2021). Feasibility and impact of a musculoskeletal health for musicians (MHM) program for musician students: A randomized controlled pilot study. J. Hand Ther..

[45] Fteiha B., Abul Al-Rub T., Schwartz E., Lachish T. (2021). Morbidity among Arab-Israeli and Palestinian Hajj Pilgrims: A Prospective Study. Am. J. Trop. Med. Hyg..

[46] Tabatabaei A., Mortazavi S.M., Shamspour N., Shushtarizadeh N. (2015). Health knowledge, attitude and practice among Iranian pilgrims. Iran. Red. Crescent. Med. J..

[47] Alhajri W., Templeton A., Moore A. (2023). Social norms and risks at mass gatherings: A systematic review. Int. J. Disaster Risk Reduct..

[48] Alqahtani A.S., Sheikh M., Wiley K., Heywood A.E. (2015). Australian Hajj pilgrims’ infection control beliefs and practices: Insight with implications for public health approaches. Travel Med. Infect. Dis..

[49] Alshammari S., Ba-Aoum M., Alganmi N., Showail A. (2025). Modeling Infectious Disease Epidemics in Mass Religious Gatherings: A Systematic Review. ACM Trans. Model. Comput. Simul..

[50] World Health Organization (2007). Effective Media Communication During Public Health Emergencies: A WHO Handbook.

[51] Das T., Holland P., Ahmed M., Husain L. (2021). Sustainable development goal 3: Good health and well-being. South-East Asia Eye Health: Systems, Practices, and Challenges.

[52] Elendu C., Amaechi D.C., Okatta A.U., Amaechi E.C.M., Elendu T.C.B., Ezeh C.P.M., Elendu I.D.B. (2024). The impact of simulation-based training in medical education: A review. Medicine.

[53] Chandran V.P., Balakrishnan A., Rashid M., Kulyadi G.P., Khan S., Devi E.S., Nair S., Thunga G., Rahman M.S. (2022). Mobile applications in medical education: A systematic review and meta-analysis. PLoS ONE.

[54] Fitzpatrick P.J. (2023). Improving health literacy using the power of digital communications to achieve better health outcomes for patients and practitioners. Front. Digit. Health.

[55] Dwivedi Y.K., Ismagilova E., Hughes D.L., Carlson J., Filieri R., Jacobson J., Jain V., Karjaluoto H., Kefi H., Krishen A.S. (2021). Setting the future of digital and social media marketing research: Perspectives and research propositions. Int. J. Inf. Manag..

[56] Alsulami A., Sacgaca L., Pangket P., Pasay-An E., Al Amoudi F.A., Alreshidi M.S., Alrashedi N., Mostoles R., Buta J., Areola B. (2025). Exploring the Relationship Between Knowledge, Attitudes, Self-Efficacy, and Infection Control Practices Among Saudi Arabian Nurses: A Multi-Center Study. Healthcare.

[57] Árvavölgyi B., Sági J. (2019). Pilgrimage and its perception in a local religious community. Int. J. Relig. Tour. Pilgr..

[58] Albutti A., Mahdi H.A., Alwashmi A.S., Alfelali M., Barasheed O., Barnes E.H., Shaban R.Z., Booy R., Rashid H. (2024). The relationship between hand hygiene and rates of acute respiratory infections among Umrah pilgrims: A pilot randomised controlled trial. J. Infect. Public Health.

[59] Allegranzi B., Storr J., Dziekan G., Leotsakos A., Donaldson L., Pittet D. (2007). The first global patient safety challenge “clean care is safer care”: From launch to current progress and achievements. J. Hosp. Infect..

[60] Goni M.D., Hasan H., Wan-Arfah N., Naing N.N., Deris Z.Z., Arifin W.N., Baaba A.A., Aliyu A., Adam B.M. (2020). Health Education Intervention as an Effective Means for Prevention of Respiratory Infections Among Hajj Pilgrims: A Review. Front. Public Health.

[61] Qualls N. (2017). Community Mitigation Guidelines to Prevent Pandemic Influenza—United States, 2017.

[62] Getz D., Page S.J. (2016). Progress and prospects for event tourism research. Tour. Manag..

[63] Laing R., Hogerzeil H., Ross-Degnan D. (2001). Ten recommendations to improve use of medicines in developing countries. Health Policy Plan..

[64] Williams J.L., Walker R.J., Smalls B.L., Campbell J.A., Egede L.E. (2014). Effective interventions to improve medication adherence in Type 2 diabetes: A systematic review. Diabetes Manag..

[65] Wee J., Tan X.R., Gunther S.H., Ihsan M., Leow M.K.S., Tan D.S.-Y., Eriksson J.G., Lee J.K.W. (2023). Effects of Medications on Heat Loss Capacity in Chronic Disease Patients: Health Implications Amidst Global Warming. Pharmacol. Rev..

[66] Baomer A.A., el Bushra H.E. (1998). Profile of diabetic Omani pilgrims to Mecca. East Afr. Med. J..

[67] Chan C., Ackermann B. (2014). Evidence-informed physical therapy management of performance-related musculoskeletal disorders in musicians. Front. Psychol..

[68] Araújo L.S., Wasley D., Redding E., Atkins L., Perkins R., Ginsborg J., Williamon A. (2020). Fit to Perform: A Profile of Higher Education Music Students’ Physical Fitness. Front. Psychol..

[69] Rosset M., Baumann E., Altenmüller E. (2022). A Longitudinal Study of Physical and Mental Health and Health-Related Attitudes Among Music Students: Potentials and Challenges for University Health Promotion Programs. Front. Psychol..

[70] Rodwin A.H., Shimizu R., Travis R., James K.J., Banya M., Munson M.R. (2022). A Systematic Review of Music-Based Interventions to Improve Treatment Engagement and Mental Health Outcomes for Adolescents and Young Adults. Child Adolesc. Soc. Work J..

[71] Rahman M.M., Al-Zahrani S., Al-Qattan M.M. (1999). “Outbreak” of hand injuries during Hajj festivities in Saudi Arabia. Ann. Plast. Surg..

