# Supplementary material for: Health Education in Mass Gatherings: A Scoping Review to Guide Public Health Preparedness and Practice
Source: Healthcare (Basel). 2025 Aug 7;13(15):1926. doi: 10.3390/healthcare13151926 (PMC12346179; doi:10.3390/healthcare13151926)
Supplement: Supplementary file 1 [file healthcare-13-01926-s001.zip › healthcare-3704376-supplementary.pdf]

Table of Contents

Supplementary file S1: The search strategy in databases *2*

S1A: PubMed 2

S1B: Scopus 2

S1C: Embase 3

S1D: Cochrane 3

Supplementary file S2: The list of excluded studies with reasons *4*

Supplementary file 3: The detailed contents of health education *12*

Supplementary Sile S1: The search strategy in databases

S1A: PubMed

Search number	Query	Results
**1**	(health education[Title/Abstract] OR school based intervention[Title/Abstract] OR education[Title/Abstract] OR medical education[Title/Abstract] OR educational video[Title/Abstract] OR video learning[Title/Abstract] OR e-learning[Title/Abstract]) OR (lecture[Title/Abstract]) OR "Health Education (MeSH)" OR "Health Promotion (MeSH)" OR Health (promot* OR knowledge* OR practice* OR educat*) OR Planning OR Prevention OR "Health services	714,229
**2**	mass gathering*[Title/Abstract] OR mass event*[Title/Abstract] OR crowd[Title/Abstract] OR hajj[Title/Abstract] OR Umrah[Title/Abstract] OR festival[Title/Abstract] OR concert[Title/Abstract] OR pilgrimage[Title/Abstract] OR social event[Title/Abstract] OR "crowd management" OR "crowd*" OR "event" OR "festival*" OR "game*" OR "holiday*" OR "mass gathering" OR "music" OR "natural event" OR "parade" OR "public events" OR "religious" OR "school events" OR "sport events" OR "sports" OR "university events"	29,350
**3**	#1 AND #2	674

S1B: Scopus

(TITLE-ABS-KEY ("mass gathering*" OR "mass event*" OR "crowd" OR "hajj" OR "umrah" OR "festival" OR "concert" OR "pilgrimage" OR "social event")) AND (TITLE-ABS-KEY ("health education" OR "medical education" OR "health educational video" OR "health learning" OR "health e-learning" OR "health lecture" OR "medical lecture")) AND (LIMIT-TO (DOCTYPE , "ar")) — 445 results

Show less

(TITLE-ABS-KEY ("mass gathering*" OR "mass event*" OR "crowd" OR "hajj" OR "umrah" OR "festival" OR "concert" OR "pilgrimage" OR "social event")) AND (TITLE-ABS-KEY ("health education" OR "medical education" OR "health educational video" OR "health learning" OR "health e-learning" OR "health lecture" OR "medical lecture")) — 612 results

Show less

TITLE-ABS-KEY ("health education" OR "medical education" OR "health educational video" OR "health learning" OR "health e-learning" OR "health lecture" OR "medical lecture") — 508,292 results

Show less

TITLE-ABS-KEY ("mass gathering*" OR "mass event*" OR "crowd" OR "hajj" OR "umrah" OR "festival" OR "concert" OR "pilgrimage" OR "social event") — 129,722 results

Show less

S1C: Embase

History | Save | Delete | Print view | Export | Email | Combine | using And / Or | Collapse

#8	#7 AND 'article'/it	571
#7	#3 AND #6	925
#6	#4 OR #5	862,507
#5	'health education' OR 'medical education'	594,031
#4	'health education'/exp OR 'health education'	463,739
#3	#1 OR #2	41,639
#2	'mass gathering*' OR 'mass event*' OR 'crowd' OR 'hajj' OR 'umrah' OR 'festival' OR 'concert' OR 'pilgrimage' OR 'social event'	41,639
#1	'mass gathering'/exp OR 'mass gathering'	1,261

S1D: Cochrane

Search | Search manager | Medical terms (MeSH) | PICO search

Save this search | View/Share saved searches | ? Search help

Print search history

#1	(mass gathering):ti,ab,kw (Word variations have been searched)	S	Limits	390
#2	("Hajj" OR "Umrah" OR "Concert" OR "Social event" OR "mass gathering"):ti,ab,kw (Word variations have been searched)	S	Limits	860
#3	#1 OR #2		Limits	1232
#4	("Health education" OR "Medical education"):ti,ab,kw (Word variations have been searched)	S	Limits	20011
#5	#3 AND #4		Limits	40

Supplementary Sile S2: The list of excluded studies with reasons

Number	Title	Reason
1	Two's company, three hundred million's a crowd: balancing clinical integrity and population consciousness in medical education	Case studies/short communications/ editorials/guidelines
2	Commentary: improving medical education during financially challenging times	Case studies/short communications/ editorials/guidelines
3	Carriage of Neisseria meningitidis Among Umrah Pilgrims: Circulating Serogroups and Antibiotic Resistance	No Health Education
4	Barriers to and Facilitators of Asthma Care For Malaysian Hajj Pilgrims: A Qualitative Study	qualitative study
5	A Master of Public Health with a Concentration in Mass Gatherings Health	Not outcome of interest
6	Drug-related deaths at Australian music festivals	No Health Education
7	The Extent of Medication Errors During Hajj in the Kingdom of Saudi Arabia	No Health Education
8	Recommendations for management of diabetes and its complications during Hajj (Muslim pilgrimage)	Review
9	Hepatitis B Screening and Awareness in the Milwaukee Hmong Community	Not mass gathering
10	An analysis of use of crowd medical services at an English football league club	No Health Education
11	Influenza vaccination among Saudi Hajj pilgrims: Revealing the uptake and vaccination barriers	No Health Education
12	Mass-Gathering Medical Care Provided by a Collegiate-Based First Response Service at an Annual College Music Festival and Campus-Wide Celebration	Case studies/short communications/ editorials/guidelines
13	Tuberculosis knowledge, attitude and practice among healthcare workers during the 2016 Hajj	No Health Education
14	Protective practices and respiratory illness among US travelers to the 2009 Hajj	No Health Education
15	Disparities of demographics, clinical characteristics, and hospital outcomes of AMI pilgrims vs non-pilgrims-tertiary center experience	Case studies/short communications/ editorials/guidelines
16	Understanding illnesses through a film festival: An observational study	Not mass gathering
17	An interventional program for nursing staff on selected mass gathering infectious diseases at Hajj	Not outcome of interest
18	Knowledge, attitude and practice (KAP) survey regarding antibiotic use among pilgrims attending the 2015 Hajj mass gathering	No Health Education
19	Diabetes care during Hajj	Case studies/short communications/ editorials/guidelines
20	Hyperglycaemic and hypoglycaemic emergencies among patients with diabetes mellitus who participated in pilgrims of the 2019/1440H Hajj season	No Health Education
21	Exploring the experiences, motivations, and skillsets of nurse volunteers during Hajj: implications for enhancing volunteer programs	No Health Education

22	Knowledge, Attitude and Practice (KAP) Survey Concerning Antimicrobial Use among Australian Hajj Pilgrims	No Health Education
23	An Effective Risk Minimization Strategy Applied to an Outdoor Music Festival: A Multi-Agency Approach	No Health Education
24	Noncommunicable Disease Emergencies During Arbaeenia Mass Gathering at Public Hospitals in Karbala, Najaf, and Babel Governorates, Iraq, 2014: Cross-Sectional Study	No Health Education
25	Emergency nurse disaster preparedness during mass gatherings: a cross-sectional survey of emergency nurses' perceptions in hospitals in Mecca, Saudi Arabia	No Health Education
26	Respiratory Tract Infections and its Preventive Measures among Hajj Pilgrims, 2010: A Nested Case Control Study	No Health Education
27	Awareness that cocaine can contain fentanyl among nightclub and festival attendees in New York City, 2018-2022	No Health Education
28	Determinants of tetanus, diphtheria and poliomyelitis vaccinations among Hajj pilgrims, Marseille, France	No Health Education
29	Pilgrims satisfaction with ambulatory health services in Makkah, 2008	No Health Education
30	Hyperglycemic emergencies in Indian patients with diabetes mellitus on pilgrimage to Amarnathji yatra	No Health Education
31	Safety preparedness of urban community for New Year fireworks in Tehran	Not mass gathering
32	Outbreak of salmonellosis associated with consumption of pulled pork at a church festival - Hamilton County, Ohio, 2010	Case studies/short communications/ editorials/guidelines
33	Incidence of Hajj-associated febrile cough episodes among French pilgrims: a prospective cohort study on the influence of statin use and risk factors	No Health Education
34	Medical care at mass gatherings: emergency medical services at large-scale rave events	No Health Education
35	Developing an enhanced minor injury unit for support of urban festivities	No Health Education
36	Simulated-Scenario and Peer-Mentorship Curriculum to Train Prehospital Providers in the Practice of Mass Gathering Medicine	No Health Education
37	Hospital preparedness for mass gathering events and mass casualty incidents in Matera, Italy, European Capital of Culture 2019	Not outcome of interest
38	Infection transmission via mobile phones in a mass gathering setting: Public knowledge, attitudes and practices in Makkah city, Saudi Arabia	No Health Education
39	Knowledge, Attitude, and Practice Regarding Antibiotic Use and Resistance for Upper Respiratory Tract Infections among the Population Attending a Mass Gathering in Central India: A Cross-Sectional Study	No Health Education
40	Knowledge, Attitude, and Practices of Food Handlers on Food Safety and Personal Hygiene During Arbaeenia Mass Gathering, Baghdad, Iraq, 2014: Cross-Sectional Study	No Health Education
41	Spectator medicine at an international mega sports event: Rugby World Cup 2019 in Japan	No Health Education
42	Mass-gathering medical care: retrospective analysis of patient presentations over five years at a multi-day mass gathering	Case studies/short communications/ editorials/guidelines
43	Challenges and Opportunities to Maximize Mental Health among Shipboard Sailors: A Qualitative Study	Not outcome of interest

44	Impact of educational intervention on understanding health recommendations after liver transplantation	Not mass gathering
45	Uniting hearts and minds: experiences from a pilot festival of youth creative expressions on mental health in India	Not mass gathering
46	Exploring attitudes towards health preparedness in the Middle East and North Africa against chemical, biological, radiological, and nuclear threats: A qualitative study	qualitative study
47	Factors influencing uptake of schistosomiasis research findings in ingwavuma area, uMkhanyakude District, Kwazulu-Natal, South Africa	Not mass gathering
48	Analysis of Healthcazre Services in 2019 Arbaeen March: A Qualitative Study	qualitative study
49	Proactive and Reactive Recruitment of Black and Latino Adolescents in a Vaping Prevention Randomized Controlled Trial	Not mass gathering
50	Assessing the impact of an orientation week on acclimation to radiology residency	Not mass gathering
51	Promoting the â€œrevitalization of healthy life expectancyâ€• with the town of anamizu	Review
52	Assessment of knowledge, attitude and practice towards prevention of respiratory tract infections among hajj and umrah pilgrims from Malaysia in 2018	No Health Education
53	Pre-travel advice for the Hungarian pilgrims of El Camino: Travel risks and instructions	Non-English
54	Mandatory meningococcal vaccine, and other recommended immunisations: Uptake, barriers, and facilitators among health care workers and trainees at Hajj	Not outcome of interest
55	Innovative community-based initiatives to engage VFR travelers	Case studies/short communications/ editorials/guidelines
56	Evaluation of the actions taken in order to increase the number of women undergoing preventive mammography and Pap smear in Kujawsko-Pomorskie province	Not mass gathering
57	Acceptance and Adverse Effects of H1N1 Vaccinations among a Cohort of National Guard Health Care Workers during the 2009 Hajj Season	No Health Education
58	Key informant interviews with coordinators of special events conducted to increase cancer screening in the United States	Not mass gathering
59	The potential for pneumococcal vaccination in Hajj pilgrims: Expert opinion	Case studies/short communications/ editorials/guidelines
60	The impact of a community-based risky drinking intervention (Beat da Binge) on Indigenous young people Health behavior, health promotion and society	Not mass gathering
61	Development of a disaster preparedness curriculum for medical students: a pilot study of incorporating local events into training opportunities	No Health Education
62	Concert Outreach: A Creative Approach to Adolescent HIV Education	Not mass gathering
63	Uganda takes the AIDS drama to its schools.	Not mass gathering
64	How do communities want their information? Designing educational outreach on organ donation for Asian Americans	Not mass gathering
65	Investigation on health education level in major crowds in areas difficult to prevent and cure IDD in Xinjiang	Not mass gathering
66	The race to beat AIDS on the Lao-Thai border.	No Health Education

67	Health promotion and disease prevention	Case studies/short communications/ editorials/guidelines
68	Successful approaches. Educating through entertainment, theatre and AIDS.	Not mass gathering
69	Evaluation of a community-based health education program for salt reduction through media campaigns	Non-English
70	The cancer update campaign in Western Australia - Informing the community, influencing the agenda and helping the bottom line	Not mass gathering
71	Strategies against the stigmatisation of mentally ill subjects and their practical realisation in the example of irrsinnig menschlich e.V.	Not mass gathering
72	Administration and evaluation of rural health services. I. A tetanus control program in Haiti	Not mass gathering
73	Evaluation of the international garden festival health fair	Not mass gathering
74	Great British Fun Run	Case studies/short communications/ editorials/guidelines
75	Epidemiology and response to the COVID-19 pandemic in the Dadaab Refugee Camp Complex, Kenya, March 2020â€“December 2022	No Health Education
76	Stress induced immunomodulation and the risk of infections during major sporting events: Implications for the FIFA World Cup 2022	Case studies/short communications/ editorials/guidelines
77	Community engagement and social participation in dengue prevention: A cross-sectional study in Dhaka City	Not mass gathering
78	Unintended consequences of implementing non-pharmaceutical interventions for the COVID-19 response in Africa: experiences from DRC, Nigeria, Senegal, and Uganda	Not mass gathering
79	COVID-19 Knowledge and Prevention Behaviors in Rural Zambia: A Qualitative Application of the Information-Motivation-Behavioral Skills Model	Not mass gathering
80	Young Adult Responses to Peer Crowd-Based Targeting in E-cigarette Advertisements: An Experimental Study	Not mass gathering
81	Novel intervention to promote COVID-19 protective behaviours among Black and South Asian communities in the UK: protocol for a mixed-methods pilot evaluation	Case studies/short communications/ editorials/guidelines
82	Effect of Educational Video on Maternal Nutrition, Hygiene and Sanitation among Maternal Age Group Women: A Prospective Interventional Study from Prayagraj, Uttar Pradesh, India	Not mass gathering
83	Wastewater surveillance as an epidemiological tool at mass gathering events: A FIFA World Cup Qatar 2022 perspective and implication to other mass gathering events	No Health Education
84	Hygiene and Health Coaching for Community Readiness to Perform the Hajj during an Ongoing COVID-19 Pandemic	Not outcome of interest
85	Post-migration food habits of New Zealand South Asian migrants: Implications for health promotion practice	Not mass gathering
86	Cholera vaccine and mass gatherings: protecting the crowds	No Health Education
87	The Trauma Challenges in the Arbaeen Ceremony	Not mass gathering
88	A Malaysian consensus recommendation for the prevention of influenza in older persons	Case studies/short communications/ editorials/guidelines

89	Assessment of an intensive education program for pharmacists on treatment of tobacco use disorder using an objective structured clinical examination: a randomized controlled trial	No Health Education
90	Decisions to attend holiday gatherings during COVID-19 and engagement in key prevention strategies: United States, January 2021	No Health Education
91	Iraq experience in handling the COVID-19 pandemic: implications of public health challenges and lessons learned for future epidemic preparedness planning	Review
92	Effectiveness of educational intervention regarding knowledge of Self-care management of Diabetes Mellitus during Pandemic COVID 19 among senior citizens residing in selected old age homes	Not mass gathering
93	Knowledge, Attitude, and Perception of Health Care Personnel Working in Intensive Care Units of Mass Gatherings Toward the Application of Telemedicine Robotic Remote-Presence Technology: A Cross-Sectional Multicenter Study	No Health Education
94	STI testing among young people attending music festivals in New South Wales, Australia: Exploring the client segmentation concept in the 'Down to Test' program	No Health Education
95	Living with COVID-19: mass gatherings and minimizing risk	Case studies/short communications/ editorials/guidelines
96	COVID-19 prevention protocol for an organised mass gathering-an essential requisite in pandemic and post-pandemic phase	No Health Education
97	Health risks, preventive behaviours and respiratory illnesses at the 2019 arbaeen: Implications for covid-19 and other pandemics	No Health Education
98	Capturing the wisdom of the crowd: health professions' educators meet at aÂ virtual world cafÃ©	Not mass gathering
99	Insight into COVID-19 responses and initiatives from Pakistan	Not mass gathering
100	Analysis on cluster cases of COVID-19 in Tianjin	Not mass gathering
101	A Diabetes Screening and Educational Event in Rural Alabama	Not mass gathering
102	Saudi Arabia's drastic measures to curb the COVID-19 outbreak: Temporary suspension of the Umrah pilgrimage	Review
103	Repercussions of mass gathering: Covid-19 pandemic	No Health Education
104	Effect of Face-to-Face vs Virtual Reality Training on Cardiopulmonary Resuscitation Quality: A Randomized Clinical Trial	Not mass gathering
105	A crowdsourcing open contest to design pre-exposure prophylaxis promotion messages: Protocol for an exploratory mixed methods study	Not mass gathering
106	Why are you running and does it hurt? Pain, motivations and beliefs about injury prevention among participants of a large-scale public running event	No Health Education
107	Knowledge, attitude and practice of pilgrims regarding heat-related illnesses during the 2017 hajj mass gathering	No Health Education
108	Innovative preventive and resilience approaches against Aedes-linked vector-borne arboviral diseases threat and epidemics burden in gulf council countries	review
109	Analysis of quality assurance on health development of hajj pilgrims in Bojonegoro district	No Health Education
110	Peer crowd segmentation for targeting public education campaigns: Hip hop youth and tobacco use	No Health Education

111	Building community resilience: A scalable model for hemorrhage-control training at a mass gathering site, using the RE-AIM framework	No Health Education
112	Investigating hajj as favourable time for smoking cessation	No Health Education
113	The community benefits of organizing a public health film festival at a school of public health	Case studies/short communications/ editorials/guidelines
114	Respiratory viruses in returning Hajj & Umrah pilgrims with acute respiratory illness in 2014-2015	No Health Education
115	Rhythms of the Heart: An interprofessional community health collaboration to increase cardiovascular health knowledge	Case studies/short communications/ editorials/guidelines
116	Alcohol and injury risk at a Western Australian school Leavers Festival	Not mass gathering
117	Menstrual abnormalities amongst female South African Hajj pilgrims: a cross-sectional study	No Health Education
118	Developing religiously-tailored health messages for behavioral change: Introducing the reframe, reprioritize, and reform (â€œ3Râ€•) model	Not mass gathering
119	Developing Public Health Initiatives through Understanding Motivations of the Audience at Mass-Gathering Events	Case studies/short communications/ editorials/guidelines
120	Exploring challenges of health system preparedness for communicable diseases in Arbaeen mass gathering: A qualitative study.	qualitative study
121	Estimating the frequency and characteristics of respiratory disease outbreaks at mass gatherings in the United States: Findings from a state and local health department assessment	Not outcome of interest
122	Assessment of knowledge, practice and barrier in use of facemask among university students	Not mass gathering
123	Raising awareness of health care providers about MERSCoV infection in public hospitals in Mecca, Saudi Arabia	Not outcome of interest
124	Mozambique field epidemiology and laboratory training program: A pathway for strengthening human resources in applied epidemiology	No Health Education
125	Sports Day in Canada: examining the benefits for event organizers (2010â€“2013)	Not mass gathering
126	A career in a Kingdom: Journeys in infection, Mass Gathering Medicine and public health diplomacy	Case studies/short communications/ editorials/guidelines
127	Steps Toward Creating A Therapeutic Community for Inpatients Suffering from Chronic Ulcers: Lessons from Allada Buruli Ulcer Treatment Hospital in Benin	Not mass gathering
128	Prevention of meningococcal disease during the Hajj and Umrah mass gatherings: Past and current measures and future prospects	Not outcome of interest
129	The annual Hajj pilgrimageâ€”minimizing the risk of ill health in pilgrims from Europe and opportunity for driving the best prevention and health promotion guidelines	Case studies/short communications/ editorials/guidelines
130	Adding a psychological dimension to mass gatherings medicine	No Health Education
131	Hypertension and Diabetes Mellitus: A Preliminary South African Health Promotion Activity Using Service-Learning Principles	Not mass gathering

132	Knowledge, attitudes and practices concerning Middle East respiratory syndrome among Umrah and Hajj pilgrims in Samsun, Turkey, 2015	Case studies/short communications/ editorials/guidelines
133	Medical strategies in mass gathering: The experience of the Guadalajara International Book Fair	Non-English
134	Australian Hajj pilgrimsâ€™ knowledge, attitude and perception about ebola, november 2014 to february 2015	Case studies/short communications/ editorials/guidelines
135	Increase in use of protective earplugs by Rock and Roll concert attendees when provided for free at concert venues	Case studies/short communications/ editorials/guidelines
136	Health warnings about obesity: Kingâ€™s Festival of Food and Ideas	Case studies/short communications/ editorials/guidelines
137	Has Hajj-associated Middle East Respiratory Syndrome Coronavirus transmission occurred? The case for effective post-Hajj surveillance for infection	Case studies/short communications/ editorials/guidelines
138	Improvement of Hajj pilgrims' health status in Hajj-e-Tamattu	No Health Education
139	Tackling malaria, village by village: A report on a concerted information intervention by medical students and the community in Mifumi, Eastern Uganda	Not mass gathering
140	Fireworks injuries in children: A prospective study during the festival of lights	Not mass gathering
141	Good practices and health policy analysis in European sports stadia: results from the 'Healthy Stadia' project	No Health Education
142	A review of Ghana's 2009-2013 integrated strategic response plan for pandemic influenza: Illustrative study of the perceived adequacy of preparedness for the pandemic influenza of sub-Sahara Africa	Not mass gathering
143	HIV/AIDS: Preliminary health promotion activity based on service-learning principles in Grahamstown, Eastern Cape, South Africa	Not mass gathering
144	Public health preparedness for the world's largest mass gathering: 2010 world exposition in Shanghai, China	Case studies/short communications/ editorials/guidelines
145	Pilgrimage to wellness: an exploratory report of rural African American clergy perceptions of church health promotion capacity.	Not mass gathering
146	Building public policy to support young people in reducing alcohol-related harm when partying at Schoolies Festivals	Case studies/short communications/ editorials/guidelines
147	Active for a day: Predictors of relapse among previously active mass event participants	No Health Education
148	Infectious disease surveillance for the London 2012 Olympic and Paralympic Games	No Health Education
149	The Australian national binge drinking campaign: campaign recognition among young people at a music festival who report risky drinking.	No Health Education
150	Health conditions for travellers to Saudi Arabia for the pilgrimage to Mecca (Hajj).	Case studies/short communications/ editorials/guidelines
151	Intervention for prevention and therapy of overweight-obesity in an engineering company	Not mass gathering

152	Randomized controlled trial to increase physical activity among insufficiently active women following their participation in a mass event	No Health Education
153	Ensuring and promoting food safety during the 2008 Beijing Olympics	Case studies/short communications/ editorials/guidelines
154	Clinical analysis of firework-related ocular injuries during Spring Festival 2009	No Health Education
155	Diabetic profile of Pakistani pilgrims in Makkah during Hajj season (2007-2008)	Case studies/short communications/ editorials/guidelines
156	Using an H1N1 vaccination drive-through to introduce healthcare students and their faculty to disaster medicine.	Not outcome of interest
157	Hazards of hepatitis at the Hajj	No Health Education
158	Blessings in disguise: Public health emergency preparedness for World Youth Day 2008	review
159	OddSocks at the Melbourne Fringe Festival: A methods paper for using an arts installation in promoting public health	No Health Education
160	Factors determining use of pre-travel preventive health services by West African immigrants in the Netherlands	No Health Education
161	Usefulness of molecular techniques to identify ongoing tuberculosis transmission in Saudi Arabia	Case studies/short communications/ editorials/guidelines
162	Mass community cycling events: Who participates and is their behaviour influenced by participation?	No Health Education
163	Steady improvement of infection control services in six community hospitals in Makkah following annual audits during Hajj for four consecutive years	Not outcome of interest
164	Pediatric emergency preparedness for mass gatherings and special events	Review
165	Oro-facial injury and mouthguard usage by athletes in Nigeria	Not mass gathering
166	Travel epidemiology: The Saudi perspective	No Health Education
167	Surveillance for meningococcal carriage by Muslims returning from the Hajj to Hat Yai Airport, Thailand.	No Health Education
168	Hypoglycemia in diabetics during Hajj	Case studies/short communications/ editorials/guidelines
169	Effect of parasite screening on refugee health.	Not mass gathering
170	Outbreak' of hand injuries during Hajj festivities in Saudi Arabia	Not mass gathering
171	Profile of diabetic Omani pilgrims to Mecca	No Health Education
172	Health knowledge, attitude and practice among Iranian pilgrims	Not health education
173	Morbidity among Arab-Israeli and Palestinian Hajj Pilgrims: A Prospective Study	Not health education
174	Effectiveness of an educational program on decreasing burns and injuries in Persian festival of fire: A burden of diseases approach	Not outcome of interest
175	Feasibility and impact of a musculoskeletal health for musicians (MHM) program for musician students: A randomized controlled pilot study	Not outcome of interest

Supplementary File S3: The detailed contents of health education

Author, year	Content
Goni MD 2023	**Pre Hajj**<br>To register the biodata and medical record of the user, to educate pilgrims about infuenza and ILI prevention, to increase the perception of threats for the pilgrims by targeting the perceived susceptibility to the diseases and the perceived severity of any possible threat that can be transmitted during Hajj, to enhance the preventive practices and behavior of the pilgrims by teaching the recommended practices associated with the Hajj pilgrimage, to educate the pilgrims on cues to actions of the pilgrims regarding prevention of infuenza and ILIs,<br><br>**During Hajj**<br>Use of face mask, good hand hygiene, cough etiquette, supplements, and other health recommendations and guidelines (Dos and Don'ts), to motivate the pilgrims to take positive actions by targeting the perceived benefts and perceived barriers of the pilgrims towards infuenza and ILIs<br><br>**After Hajj**<br>Proper action to take in the event of any infection and proper medication, report any complications to the nearest medical center<br><br>**Formative assessment**<br>Through formative assessment practices, pilgrims will think at deeper levels, problem-solve effectively, ask good questions, and take ownership of their learning, using formative assessment practices on an ongoing basis helps teachers gather evidence of learning to ensure each pilgrim's instruction effectiveness, gathering evidence of learning using formative assessment practices gives teachers the information they need to tailor instruction based on students' readiness to learn
Mushi A 2021	**Training and health promotion before arrival to KSA**<br>Know where to seek medical care in KSA before arrival; know where medical mission clinics location in KSA before arrival; Hajj training received before arrival to KSA; Information about health risks in KSA before arrival about Heat related illnesses, Respiratory related illness, Gastrointestinal disorders and Information on how to seek medical help received before arrival to KSA<br>**Health messages received before arrival to KSA**<br>Hand hygiene, heat-related illnesses, cough etiquette, and others health messages received
Yezli S 2021 (2)	**Knowledge**<br>Can insulin be affected by storage temperature; Appropriate way to store unused insulin; How long can insulin be left at room temperature; How can you tell if insulin has gone bad?<br><br>**Practice**

	How did you bring your insulin to KSA; Where do you dispose of your insulin syringes (needles)
Goni MD 2021	The health education intervention module on knowledge, attitude, and practice regarding RTIs prevention among Hajj pilgrims were developed as a smartphone application through a process of consultations with a panel of experts consisting of epidemiologist, microbiologist, health educationist, computer scientist, and medical statistician delivered through a smartphone application.
Migault C 2019	The detailed the geographical area at risk of MERS-CoV contamination, the different transmission routes of the disease, symptoms, severity of the disease, availability of treatment or vaccines and preventive measures to reduce the possibility of contracting and/or spreading illness during travel and after return
Rad EH 2018	The campaign published 2000 pamphlets with various educational messages and distributed them at schools and health centers, held seven radio and television interviews in provincial networks of Guilan, installed 12 posters and banners in various regions of Rasht, Lahijan and Bandar-e- Anzali, held 2 press conferences with media, sent 70 educational and preventive messages on social networks and established a system for recording injuries and incidents during this critical festival.
Beskind LD 2017	During a commercial TV time-out at each basketball game, a 30 s Ultra-Brief CCO-CPR video (UBV) was shown on the overhead projector screen in the middle of the gymnasium that illustrated a step-by-step demonstration on how to perform CCOCPR using the three C's (Check, Call 911, Compress). All attendees who were in their seats would be able to see the video.
Wolff AL 2021	90- minute MHM workshop led by a clinician with specialization and expertise in performing arts medicine. The content for the workshops was developed based on best available evidence in collaboration with multi-disciplinary practitioners (physiatry, sports medicine, occupational therapy, and physical therapy) who are experts in performing arts medicine and music educators. The 90-minute workshop is a combination of didactic lecture (30 minutes) and a hands-on practicum (60 minutes). The didactic component included a review of common risk factors, and an explanation of the physiology of overuse injury, relevant anatomy and musculoskeletal pain conditions. The practical component includes a 60-minute discussion, demonstration, and participation in 1) practical strategies for prevention of injury in 4 categories: warm up, pacing/breaks, posture/playing position, and implementation of "smart" practice habits, and 2) specific stretches and exercises to be performed prior to playing, while onstage and during backstage breaks. Then sent weekly reminders to follow up with the strategies and recommendations,
Alamri FA et al., 2018	By training 1626 medical staff (163 doctors and 1463 technicians);who were the medical teams of 54 campaigns of 45 countries (had >4000 hajj plagiarisms). Then they educate 1,620,989 pilgrims
